# Enabling Team Autonomy in a Large Public Organization

**DOI:** 10.1007/978-3-030-58858-8_25

**Published:** 2020-08-18

**Authors:** Parastoo Mohagheghi, Casper Lassenius, Ingrid Omang Bakken

**Affiliations:** 6 IT University of Copenhagen, Copenhagen, Denmark; 7 University of British Columbia, Vancouver, BC Canada; 8 The Norwegian Labour and Welfare Administration, Oslo, Norway; 9 Simula Metropolitan Centre for Digital Engineering, Oslo, Norway; 10 Aalto University, Aalto, Finland

**Keywords:** Agile, Autonomous team, Backsourcing, Outsourcing

## Abstract

This paper describes how autonomy emerged in a team in a large public organization and which factors were important in this process. The organization has back sourced software development and abandoned a stage-based software development process with many handovers between business, IT and vendors. We collected data in four semi-structured interviews and analyzed information on changes in the structure and responsibilities of the team. The team has refined its portfolio for better cohesion, stepwise taken over the responsibility for software development from the vendor and in parallel recruited software developers, UX designers and testers. Product owners have joined the team as well. Supported by changes to the financing model, the team has transformed from mediating between business and vendors to a cross-functional product team with autonomy over its budget, backlog and software development process. As a result, the team can better balance between delivering new features and quality improvements, continuously deliver software with less overhead and focus on its mission to deliver user-friendly services with increased involvement of domain experts. Defining a clear product boundary and reducing dependencies on other teams, developing necessary skills and changing the financing model are recognized as the main success factors, as well as the main challenges in the transition process.

## Introduction

Agile software development has become the norm in the industry and is increasingly getting a foothold in the public sector, albeit so far not as an exclusive approach [1]. Public sector organizations adopt agile to solve several problems, including faster value delivery, better end-user satisfaction, better collaboration between business and IT, and cost reduction [2]. However, several factors in the government sector, such as lack of experience with agile methods, IT megaprojects and reliance on traditional procurement have been reported to make the adoption difficult [2]. When agile method adoption is combined with a change from outsourcing to insourcing, additional challenges arise such as recruiting, competence transfer and contractual negotiations [3].

In this paper, we present a single case study of a team in the Norwegian Labour and Welfare Administration (NAV) that adopted agile methods while taking ownership of previously outsourced IT systems. We describe how the team evolved from supporting product owners for the acquisition of systems from an external vendor to an autonomous agile team with full ownership of the applications it is responsible for.

## Related Work

A systematic literature review on agile methods in the public sector citing 17 primary studies reported several benefits, including faster value delivery, increased end-user satisfaction, lower cost, better collaboration between business and IT, reduced dependency on contractors, and improved team morale. Factors making adoption difficult included an unsuitable organizational culture, lack of experience with agile methods, the ingrained use of prescriptive approaches, and big bang deliveries. In addition, the public sector often runs “IT megaprojects” and relies heavily on traditional procurement and contracts, which make agile adoption challenging [2].

The 1990s and early 2000s saw a wave of outsourcing when organizations, often in the pursuit of cost savings, outsourced IT, oftentimes to low-cost countries. Lack of client involvement and competence is reported as a major challenge. A more recent trend, spurred by factors such as the recognition of IT as a core competence, unmet goals with outsourcing, and the need for better control of the IT systems, is *backsourcing (or insourcing),* i.e. bringing the outsourced components back in-house [3].

There is an extensive amount of literature on autonomous teams and different types of autonomy such as autonomy over product, people and planning decisions [4]. Autonomy has some pre-conditions, among them having the right skills in the team as well as a redundancy of skills (since it affects the team’s capability to adapt to changing situations), culture such as team orientation, sharing of information and management support in order to create the right environment for the teams [4, 5]. Team autonomy has furthermore been identified as a success factor for agile transformations [8].

## Context and Method

NAV was founded in 2006 by merging three large organizations in the public sector. NAV has 19000 employees including an IT department of over 700 employees and administers a third of the Norwegian national budget through various benefit schemes such as pension, unemployment and child-care benefits. The end-users of IT applications are twofold: organizations and individuals in Norway on the one hand, and the employees of NAV who manage the benefits on the other. Since the establishment of NAV, IT development and maintenance has mainly been outsourced to several vendors, with NAV responsible for requirements specification, acceptance testing and operation of services. In 2017, due, e.g., to high development costs and the growing need for digitalization of services, NAV decided to backsource most of the IT development. In addition, the organization has gradually adopted agile development to achieve better commitment, motivation to perform and desire for responsibility in the organization.

In previous work [7], we described a pilot study on autonomous agile teams at NAV. The experience described therein was considered successful and encouraged the organization to initiate a move towards increased cross-functionality, and to have NAV employees and vendor resources working shoulder to shoulder.

The team in this case study develops and maintains the information and user interfaces intended for the general population provided via the organization’s main website nav.no, apps and in other channels. The team is also responsible for developing organization-wide guidelines for publishing information online.

The research presented here is a single qualitative case study and part of a larger study into agile adoption and backsourcing in NAV. We selected the case due to the insights it provides on enabling team autonomy in a complex setting. We collected data through four semi-structured interviews [6], which forms the main unit of analysis. We interviewed the team leader, one product owner, a member of the team performing test, and a representative from the vendor; all being involved in the team since 2017. The interviews lasted between 60 and 90 min and were recorded and transcribed for analysis. In addition, we had a workshop with the team leader to analyze changes in the team structure and responsibilities and validated our findings with her.

## Results and Discussion

In this section, we present the results, first discussing the transition of the team, followed by discussing factors that enabled the transition towards an agile autonomous team.

### Steps in the Transition Process

Before backsourcing, over 50 applications covering a broad range of user interfaces were managed by a group of employees organized in an office in the IT department. The office managed the contract with the vendor, provided support to the business side, and followed testing, deployment, and operations of the applications. The employees of the office had roles such as functional experts, technical experts, team leaders and project leaders. Functional experts had deep domain knowledge, while technical experts focused on non-functional aspects and technology. The business side, organized in other departments in NAV, specified the requirements, prioritized the backlog, financed changes (often via projects), evaluated the estimations and design, and tested the final applications. The vendors estimated the costs of changes and designed and developed the solutions. The process thus required many handovers between business, IT and vendors. Changes were often delivered in a few large deliveries per year to manage dependencies between services.

**Fig. 1.**
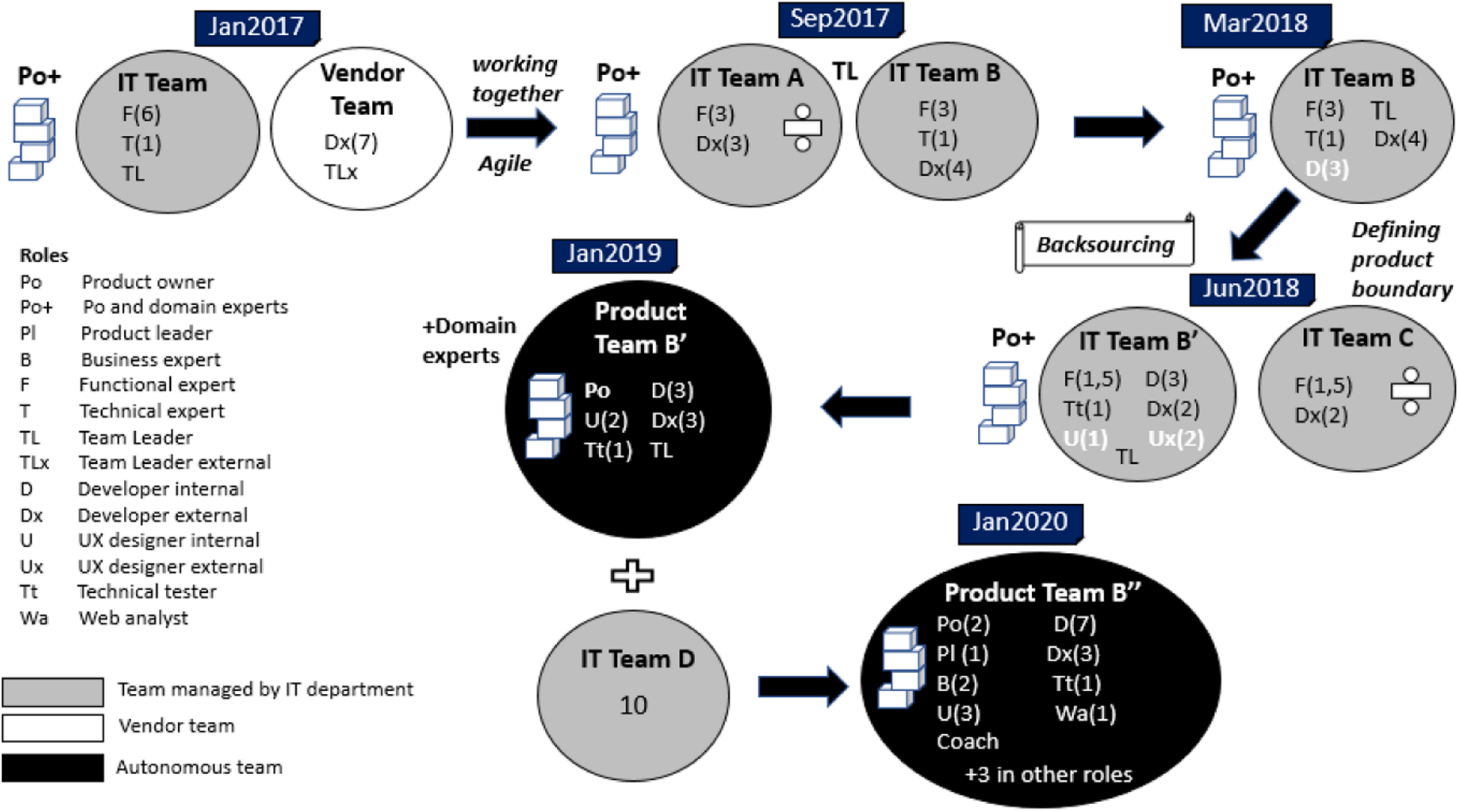
Changes in the team structure and roles for enabling team autonomy.

In the first step in the backsourcing process, the portfolio covered by the office was divided and assigned to multiple teams. In this process, the team “Self-services” was established, consisting of a team leader, six functional experts and one technical expert. The vendor had its own team collocated at NAV, with seven developers and one team leader. Figure 1 shows the changes in the team structure and roles from 2017 to 2020. The term “IT team” refers to a team managed by the IT department which has an own budget for maintenance, but depends on the business side for prioritization and financing of major changes.

The situation was changed gradually, through the following steps:

1. *Building internal development capability.* Before backsourcing, the team consisted of functional and technical experts while the developers were on the vendor side. The business department owning the applications financed recruiting 3 developers in 2018, the first one starting in February. This was considered a major step towards insourcing software development.
2. *Competence transfer*. The team had little knowledge of the code prior to the backsourcing. The IT-team and the vendor team started working together on software development for the purpose of competence transfer; including working shoulder to shoulder and pair-programming.
3. *Analyzing the applications and planning the handover.* The outsourcing contracts included steps for handover to other vendors but not to NAV. The team and the vendor performed an analysis of applications regarding their status (functionality, technical debt, security concerns and remaining failures) and developed a roadmap with milestones and actions for a stepwise handover of applications.
4. *Defining the product boundary in steps.* The old contract model put many applications to be developed by a single vendor in the same contract. As a result, the contract included over 50 applications, all related to user interfaces but managed by different stakeholders. By September 2017, the portfolio of applications was divided between two teams with a shared team leader: “Team A” (services for unemployment) and “Team B” followed here, named “Team Insight”. Some applications were handed over to other teams as well. The purpose was to separate concerns and avoid communication with multiple product owners.
5. *Transfer of ownership and responsibility; becoming self*-*sufficient competence*-*wise.* By June 2018, the team had the full responsibility for software development. A User experience (UX) designer was recruited in addition to getting support from two external UX designers. A new tester, who used to be a functional expert, was added to the team as well. Thus, the team included all necessary skills for software development. The team changed its name to “Personal users” to highlight its focus. Some functionality was left out to be handled by “Team C”.
6. *Becoming an autonomous product team.* By January 2019, the team was fully financed by the business side and one functional expert became a product owner, enhancing his competence by taking courses and participating in product owners’ fora. This type of team is called a “cross-functional product team” (in short Product Team) and the team owns its budget, product backlog and its prioritization.
7. *Enhancing the portfolio*. In January 2020, the team merged with an IT team responsible for the information on web pages, which had backsourced its applications as well (“Team D” in Fig. 1). The whole team working receives a yearly budget covering the personnel costs in full, instead of receiving funds for the changes to be implemented. The team covers two areas of functionality with team members almost 50-50 divided between these two and the possibility to assist each other when needed.

The focus of this paper has been on “Team B” and its evolution. For information, “Team A” and “Team C” are still IT Teams with some changes in their portfolio as well.

### Factors Important for Enabling Team Autonomy

The transition from an IT team mediating between product owners and vendors towards an autonomous team required several changes. We identified the following seven factors that were necessary to enable team autonomy:

1. *Full product ownership*. NAV had made a strategic decision to backsource the development of its systems and decided not to renew the contract with the vendor. Taking ownership of both the systems and the teams developing them was a precursor to creating autonomous teams. The case team has full ownership of its product and prioritizes, implements, and delivers features based on urgency and capacity.
2. *An agile mindset and way of working.* Teams can now choose their own development processes and tools, and the whole organization is developing an agile mindset, which is a profound change. The case team started to use Kanban almost overnight in September 2017. The whole team sits together and delivers continuously.
3. *Building all needed competences.* Building all the skills necessary for working autonomously was a major challenge for the organization. This included recruiting software developers in a highly competitive market, and knowledge transfer and continued collaboration with the vendor. NAV has recruited over 130 software developers since 2017 by improving its image as a high-skilled software development organization and emphasizing its role in the society. After the contract expired, a transition period was necessary for knowledge transfer and preparing the NAV team for taking charge of software development. In the team discussed here, newly recruited software developers applied pair-programming with peers from the vendor for six months. Some employees in the IT department have changed their roles and developed skills to become product owners, testers, software developers and coaches. The team leader is, e.g., now a coach for this team and other teams. The relation with the vendor was and continues to be professional with good collaboration. A new contract type is now in place to hire resources from 2–3 vendors when necessary by paying per hour.
4. *Empowerment and trust.* Without trust between the team and the surrounding organization, as well as empowerment to make and execute product and process related decisions, a team cannot function autonomously. Developing this in a large organization with a long history of traditional management can be extremely challenging.
5. *Resource*-*based financing*. The organization is gradually abandoning large projects and its traditional portfolio management process, and giving some teams, such as the team in this case their own budgets, which facilitates their autonomy.
6. *A manageable team portfolio*. The old contract model put many pieces to be developed by a single vendor in the same contract. It was necessary to focus the portfolio to reduce dependencies and give the team autonomy over the product.
7. *The right team size.* Like many organizations, NAV had challenges cutting the team size down to the optimal one, which their experience is 7–9 people, just in line with most recommendations in the team and agile literature.

### Benefits and Challenges

The team leader, product owner and the team member participating in this research reported many subjective benefits of the autonomy. The feeling of ownership and mastery had led to increased employee satisfaction. The team could now respond faster to changes since there are no handovers in the development process. Since they have product ownership, the team members can think strategically, and better balance between functional and technical improvements. This has made it possible to significantly reduce the technical debt. Cost-wise an internal employee costs less than half of an external one, and the savings are invested in new technology and in further development.

The reported challenges were mainly related to 1) the *people factor;* it was difficult to recruit enough software developers and develop skilled product owners; 2) the *product factor:* i.e. defining suitable product teams with fewer dependencies on other teams and a more coherent portfolio. In this process, it has been challenging to handover legacy applications to other teams with limited budget and capacity; and 3) the *financing model* is still not homogenous and creates challenges in prioritization and planning.

### Discussion

In this paper, we understood team autonomy in agile software development as having the power to plan and prioritize the work of the team according to budget, resources, roadmaps and constraints, and to have ownership of the processes and practices employed. This required several changes in the organizational structure and processes, and even the financing model. The autonomy to plan and prioritize work was implemented through incorporating the product ownership in the team. In this case, the organization was able to design the work of the team to have a rather independent portfolio, making it possible to have a high degree of autonomy. Our findings about how to enable team autonomy are well in line with what other cases have reported, as summarized in [8]. In particular, similar results with respect to increased morale was reported by [9, 10].

Our finding regarding the need for changing the financing model points to the importance and challenges of portfolio management in large-scale agile development, an area which currently has a lack of research. Furthermore, our findings indicate that outsourcing relationships can lead to a high degree of technical debt if there are lack of financing to remove technical debt and lack of mechanisms to incentivize high code quality.

The findings in this paper are based on four interviews with practitioners in the studied team in different roles, as well as of an analysis of other documents such as presentations. While this limits the generalizability of the findings, they are well in line with existing literature, and point toward a need for deeper understanding not only of how autonomous teams can work, but of the surrounding organizational context. Two of the authors are employees of NAV, which could introduce bias. However, the first author works in an independent role, and the findings are based on an analysis done jointly by the first two author.

## Conclusions and Future Research

We presented a case study of how team autonomy was enabled in a single team in a large public organization. We discussed that many factors are required to enable autonomy, both in the team and in the organization. The team members agreed on the benefits of the transformation that happened over the course of three years and experience increased employee satisfaction, faster response to changes and more strategic thinking.

By now, we have interviewed 35 employees in different roles and from different teams in NAV, as well as representatives from vendors. This paper is based on an initial analysis of the data from one team. We are extending our analysis to multiple teams with focus on backsourcing of software development and large-scale agile development.

We thank NAV and the interviewees for the possibility to perform the research and for sharing valuable data and insights with us.
